# A novel vibration-induced exercise paradigm improves fitness and lipid metabolism of *Caenorhabditis elegans*

**DOI:** 10.1038/s41598-018-27330-3

**Published:** 2018-06-20

**Authors:** Emelyne Teo, Krishna Chaithanya Batchu, Diogo Barardo, Linfan Xiao, Amaury Cazenave-Gassiot, Nicholas Tolwinski, Markus Wenk, Barry Halliwell, Jan Gruber

**Affiliations:** 1 NUS Graduate School for Integrative Sciences and Engineering, National University of Singapore, Singapore, Singapore; 2 Singapore Lipidomics Incubator, National University of Singapore, Singapore, Singapore; 3 Department of Biochemistry, National University of Singapore, Singapore, Singapore; 4 Science Division, Yale-NUS College, Singapore, Singapore

## Abstract

Exercise has been known to reduce the risk of obesity and metabolic syndrome, but the mechanisms underlying many exercise benefits remain unclear. This is, in part, due to a lack of exercise paradigms in invertebrate model organisms that would allow rapid mechanistic studies to be conducted. Here we report a novel exercise paradigm in *Caenorhabditis elegans (C. elegans)* that can be implemented under standard laboratory conditions. Mechanical stimulus in the form of vibration was transduced to *C. elegans* grown on solid agar media using an acoustic actuator. One day post-exercise, the exercised animals showed greater physical fitness compared to the un-exercised controls. Despite having higher mitochondrial reactive oxygen species levels, no mitohormetic adaptations and lifespan extension were observed in the exercised animals. Nonetheless, exercised animals showed lower triacylglycerides (TAG) accumulation than the controls. Among the individual TAG species, the most significant changes were found in mono- and polyunsaturated fatty acid residues. Such alteration resulted in an overall lower double bond index and peroxidation index which measure susceptibility towards lipid peroxidation. These observations are consistent with findings from mammalian exercise literature, suggesting that exercise benefits are largely conserved across different animal models.

## Introduction

Obesity and lack of physical activity (sedentary lifestyle) are growing public health problems in many developed nations^1^. Obesity increases the risk of metabolic syndromes such as hypertension, hypertriglyceridemia, blunted insulin response, elevated fasting glucose and low high-density lipoprotein cholesterol. These metabolic effects, in turn, are risk factors for cardiovascular diseases^2,3^. Exercise is one of the most effective ways to combat obesity and disorders related to inactivity^4–6^. Increasingly sedentary lifestyles are thus closely related to increases in the fraction of the population that is overweight or obese, but a lack of physical activity is harmful beyond its immediate effect on weight and is associated with an increased risk of cancer and cardiovascular diseases^7,8^. Despite these risks, many individuals find it hard to implement effective exercise regimes or comply with guidelines regarding physical activity.

A deeper understanding on how exercise promotes metabolic health would allow more targeted and effective exercise guidelines to be developed^9–11^. Such insight might even allow development of drugs or compounds that could mimic some of the key benefits associated with exercise. For example, several pharmacological agonists of mitochondrial biogenesis pathways (GW1516, GSK4716 and AICAR) have been suggested as potential exercise mimetics^12^. Exercise can serve as a powerful intervention for aspects of ageing and age-dependent disease so this exciting area of research would benefit from a deeper understanding of the molecular and biochemical mechanisms underlying exercise benefits^13,14^.

It is clear that exercise reduces obesity and positively impacts a range of related disorders in part through regulating lipid metabolism and modification of plasma lipid profiles^15,16^. However, many questions regarding the molecular and biochemical mechanisms especially of how exercise alters lipid profile and promotes health remain. While several exercise studies have profiled Triacylglyceride (TAG), lipoproteins and phospholipids, there hasn’t been a comprehensive lipidomics investigation at a detailed molecular species level^17,18^. This is, in part, due to a lack of exercise paradigm for invertebrate model organism that would allow mechanistic studies to be conducted more rapidly. A suitable exercise paradigm in simple model organisms would also allow routine analysis of tissue samples, something that cannot be readily done with human volunteers.

To circumvent the drawbacks of human studies, one common approach is to use invertebrate models^19–21^. *Caenorhabditis elegans (C. elegans)* is one such invertebrate model organism routinely used for ageing, developmental and neurobiology research. It has several unique advantages for research, including short lifespan, genetic tractability and the presence of well-characterized age-associated biochemical and behavioural phenotypes^20,22,23^. Key lipid metabolic pathways and regulators are conserved between *C. elegans* and mammals^24,25^. Recently, *C. elegans* has been used as a model of benefits associated with physical exercise. Two independent groups have developed different exercise protocols based on forced swimming of *C. elegans*^26,27^. While these paradigms have demonstrated the feasibility of using *C. elegans* to study key mechanisms underlying exercise benefits, the use of swimming as an exercise imposes several limitations. First, some mutant strains exhibit impaired swim abilities, which makes it hard to investigate the effects of exercise. Furthermore, most research in *C. elegans* is carried out on solid Nematode Growth Medium (NGM) agar plates. Swimming in liquid buffer may also induce osmotic adaptation and changes independent of exercise. Therefore, having a complementary exercise paradigm for *C. elegans*, ideally based on standard NGM would allow comparison and verification of results obtained in liquid medium.

Here, we present a novel exercise paradigm for *C. elegans* that can be implemented on standard solid NGM agar plates. We employed an acoustic actuator to deliver vibration stimuli to animals moving freely on NGM plates. This vibration stimulates animals to move more rapidly and thereby significantly increases their physical activities. This increase in activity results in adaptive changes including increased physical fitness and loss of storage lipids and changes in lipid profiles, thus suggesting adaptive changes and benefits consistent with an efficient exercise regimen. We have optimized this exercise protocol to prevent animals from habituating to the vibration stimulus. Using vibration-induced exercise, we investigated what exercise benefits could be observed in the exercised animals.

## Results

### Set up and optimization of the vibration-induced exercise paradigm

**Figure 1:**
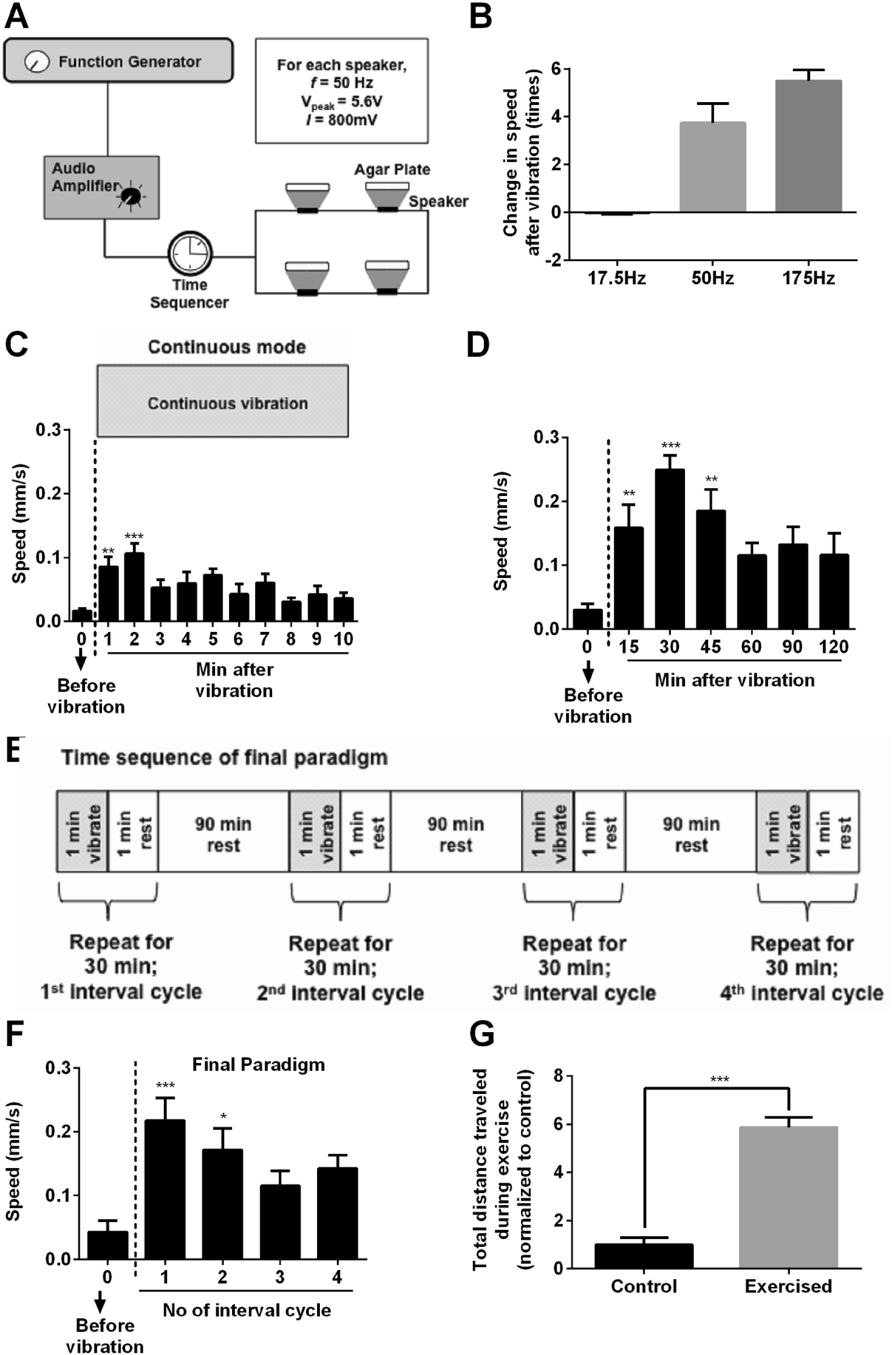
Overview of the exercise paradigm. (**A**) Block diagram of the nematode exercise system. Exercise was implemented at 25 °C. (**B**) Frequency optimization. Change in speed of animals was calculated by taking the ratio of speed before and immediately after a one min vibration to the initial speed of animal (n = 5 animals per data point). (**C**) Speed-time profile of animals during continuous vibration (**P < 0.01, ***P < 0.001; one-way ANOVA post-test Dunnett’s multiple comparisons to t = 0; n = 8 animals per data point). (**D**) Speed-time profile of animals during interval vibration mode (**P < 0.01, ***P < 0.001; one-way ANOVA post-test Dunnett’s multiple comparisons to t = 0; n = 8 animals per data point). (**E**) Schematic of the time sequence used in our final exercise paradigm. (**F**) Speed-time profile of animals during the final exercise paradigm (*P < 0.05, ***P < 0.001; one-way ANOVA post-test Dunnett’s multiple comparisons to t = 0; n = 8 animals per data point). (**G**) Total exercise effect estimation (***P < 0.001; n = 8 animals per group). Total distance travelled was calculated by taking the area under the curve of speed-time graphs during the training phase only. Speed of animals was monitored at the 1^st^ and 30^th^ minute of each interval (representing the start and end of each cycle). All experiments described in this panel were performed in JK1107 animals at day 6 post bleaching. Experiments were carried out in at least two other trials, with similar trends observed.

In order to induce exercise in *C. elegans*, we used a mechanical stimulus (vibration) to increase locomotion on solid nematode growth medium (NGM) plate. Vibration of the whole NGM surface simultaneously stimulates all the touch receptors distributed along the animal’s body surface. This has previously been shown to cause them to accelerate in their original direction or to begin moving if they are at rest^28^. To transduce vibration in a controllable manner, we used an audio speaker system connected to a function generator and audio amplifier. This set up can be used to transmit vibration at arbitrary frequency and amplitude to the animals on agar plate (Fig. 1A). One challenge with this approach is that it depends on behavioural changes in response to vibration, which are known to be subject to habituation^29^.

To overcome habituation, we carefully optimized the frequency, amplitude and sequences of stimulation used (interval/continuous) of vibration to achieve a sustained increase in physical activity required for an exercise regimen. We first explored the minimum frequency needed as stimulus in order to cause a robust increase in short term locomotion (immediately post stimulation). At a fixed amplitude of 5.6 V and a peak to peak current of 0.8 A, stimulation at 17.5 Hz did not elicit any significant increase in movement compared to control as measured by speed of movement immediately after one minute of vibration (Fig. 1B). Stimulation at 50 Hz vibration frequency with identical amplitude was sufficient to elicit an approximately 400% immediate (short term) increase in locomotion of the animals. Even though at a higher frequency of 175 Hz animals moved even more vigorously, showing an approximately 600% increase in activity immediately after a one min vibration, we found detrimental lifespan effects with extended stimulation at this frequency (Fig. S1). To prevent potential harmful effects at a higher frequency we chose 50 Hz as the frequency for all future experiments.

*C. elegans* are known to habituate to mechanical stimuli such as tapping, whereby they showed diminished withdrawal response to tapping upon repeated stimuli^29^. Continuous stimulation may therefore lead to a decline in response and blunting of the increase in locomotion speed during continuous vibration due to habituation to the signal. Additionally, animals could also become physically exhausted from continuous exercise and this again may diminish response upon continuous training. In order to determine the optimal exercise sequence for *C. elegans* and to overcome habituation and fatigue, we next examined the behaviour of animals exercising continuously at 50 Hz frequency and measured their locomotion speed during vibration every minute. As expected, we noticed a significant decline in locomotion speed after two minutes of continuous vibration (Fig. 1C), probably due to habituation or fatigue or both. To overcome this effect, we explored several interval-training sequences. The aim was to develop a paradigm that would cause the animals to exercise for extended periods of time resulting in a statistically significant overall increase in activity when averaged over time. We found that with a one-minute rest interval for every minute of vibration, the decline in response to vibration only set in 45 minutes after the onset of interval training (Fig. 1D). Even then, the total response only declined to approximately 80% of the peak movement rate measured after the first stimulus. This level of activity is still significantly above baseline (approximately eight times more; Fig. 1D). We finally adopted an interval-training program with training periods comprising alternating intervals of one minute of vibration with one minute of rest and lasting a total of 30 minutes. We followed each 30-minute training period by a 1.5-hour recuperation period, giving rise to a 2 h training-recuperation cycle. To evaluate physiological effects of exercise we repeated the whole training-recuperation cycle four times, resulting in an overall training program lasting 6.5 h from beginning of the first vibration stimulus to the end of the last stimulus (Fig. 1E). Even though there was still some decline in locomotion speed of the exercising animals after the second interval cycle compared to the first cycle (Fig. 1F), this approach led to a significant overall increase in average activity and distance travelled with exercised animals moving approximately six times farther than controls when averaged over the whole 6.5 h exercise program (Fig. 1G).

### Exercised animals showed increased fitness

We next investigated what fitness and health benefits, if any, were conferred on the exercised animals. In humans, exercise has been known to improve several components of physical fitness such as muscular performance and endurance, cardiovascular endurance and body composition^30,31^. To measure physical fitness in *C. elegans*, we investigated the spontaneous distance travel ability of the animals, which has been routinely used as a healthspan and muscular health indicator^32–34^. We measured spontaneous distance travel ability one day after exercise training, by analysing a one-minute trajectory of free-moving animals on a fresh plate without food, after allowing them to forage for five minutes.

**Figure 2:**
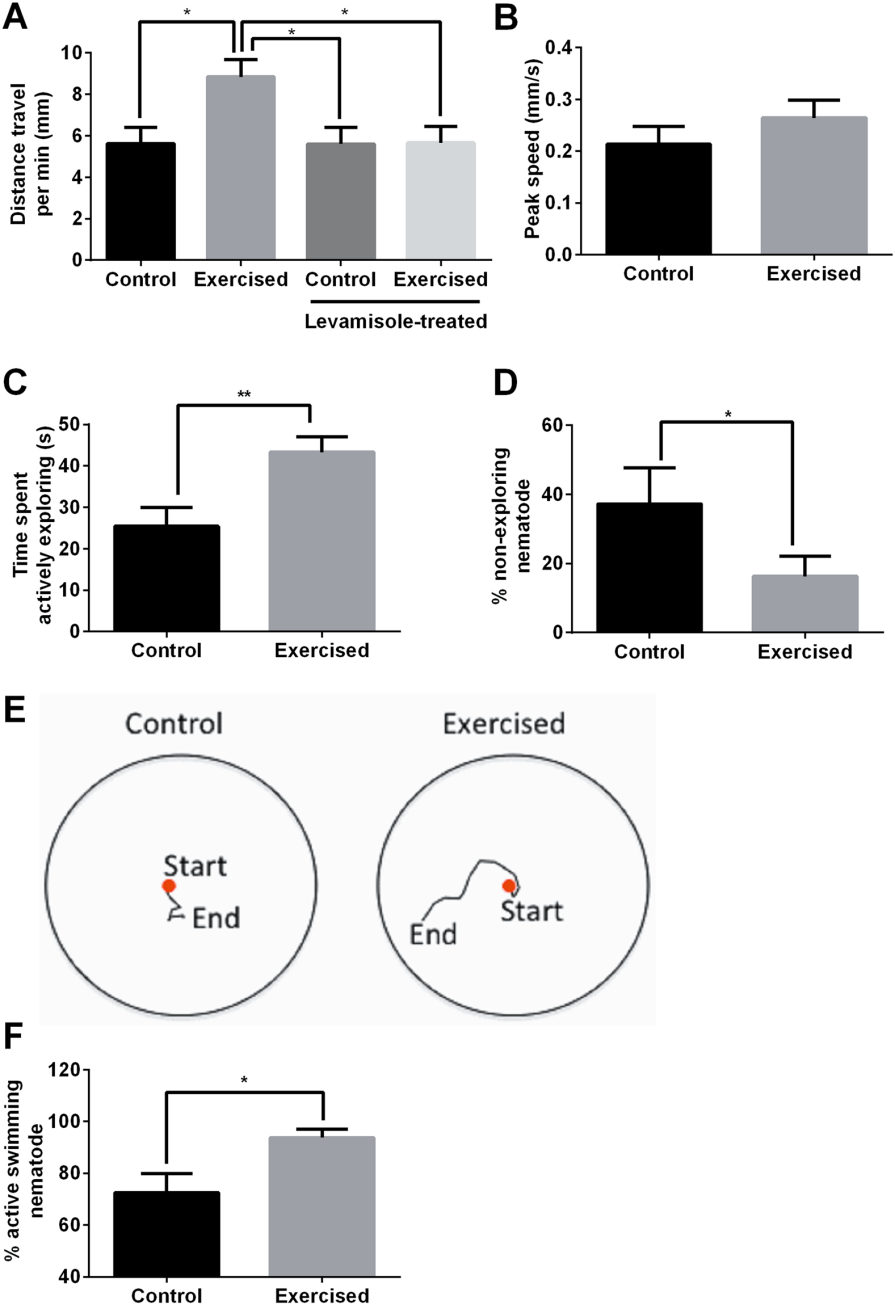
Exercise benefits observed in *C. elegans* one day post-training (day 7 post bleaching). (**A**) Exercised animals had higher spontaneous distance travel ability (*P < 0.05; n ≥ 20 animals per group). (**B**) No significant difference in peak speed of exploring was observed between exercised and control animals (n ≥ 20 animals per group). (**C**) Exercised animals spent significantly more time exploring the plate than the control animals (**P < 0.005; n ≥ 20 animals per group). (**D**) A significantly greater percentage of control animals remained close to the origin for the duration of the trial (non-exploring) compared to the exercised animals (*P < 0.05; n = 5 independent trials with at least 20 animals per group per trial). (**E**) Representative images showing a typical non-exploring control and an actively exploring exercised animal. (**F**) There was a higher percentage of actively swimming nematodes in the exercised group after being placed in M9 buffer for 2 h compared to controls (*P < 0.05; n = 10 independent trials with at least 20 animals per group per trial). All experiments described in this panel have been carried out in at least two other trials, with similar trends observed.

We found that the exercised animals voluntarily travelled a significantly greater distance during food foraging compared to the controls one day post-exercise (Fig. 2A), suggesting that they are physically more fit and active than the controls. To confirm that this fitness benefit was mediated by the enhanced activity during the vibration paradigm and not caused simply by mechanical stimulation (vibration) or reduced food intake, we used levamisole to induce transient paralysis and inhibit pharyngeal pumping, and subjected these animals to the vibration paradigm. No distance travel benefit was observed in the levamisole-treated animals subjected to the exercise paradigm (Fig. 2A), thus demonstrating that the fitness benefits are mediated by exercise (enhanced activity) rather than mechanical stimulation or starvation.

To further determine whether the greater distance travel ability of the exercised animals was due to a faster spontaneous moving speed or an increase in food foraging and exploration behaviour (i.e if animals simply spend more time actively moving), we analysed their peak movement speed and time spent actively exploring (defined as moving more than 0.4 mm in a five-second interval). There was no significant difference in the peak speed between the control and exercised animals (Fig. 2B), but the exercised animals spent twice as much time actively exploring compared to controls (Fig. 2C). In five independent experiments, we found that un-exercised animals rarely explored far from the origin, while the exercised animals explored more actively across the plate (Fig. 2D and E). These observations suggest that exercise increases food foraging and exploration behaviour of these animals.

We also conducted swimming assay for the animals as another fitness indicator, by placing the animals in M9 buffer and scored the percentage of active swimming animals two hours later. We found that one day post exercise, there was a significantly higher percentage of exercised animals that were still actively swimming after two hours in M9 buffer (Fig. 2F), suggesting that the exercise regimen improves swimming endurance of *C. elegans*. Given that this swimming behaviour is regulated by Acetylcholine and GABA neurotransmitters^35^, the increased swim endurance of the exercised animals may also be mediated partly by neuronal adaptations.

### Exercised animals showed no consistent lifespan enhancement

**Figure 3:**
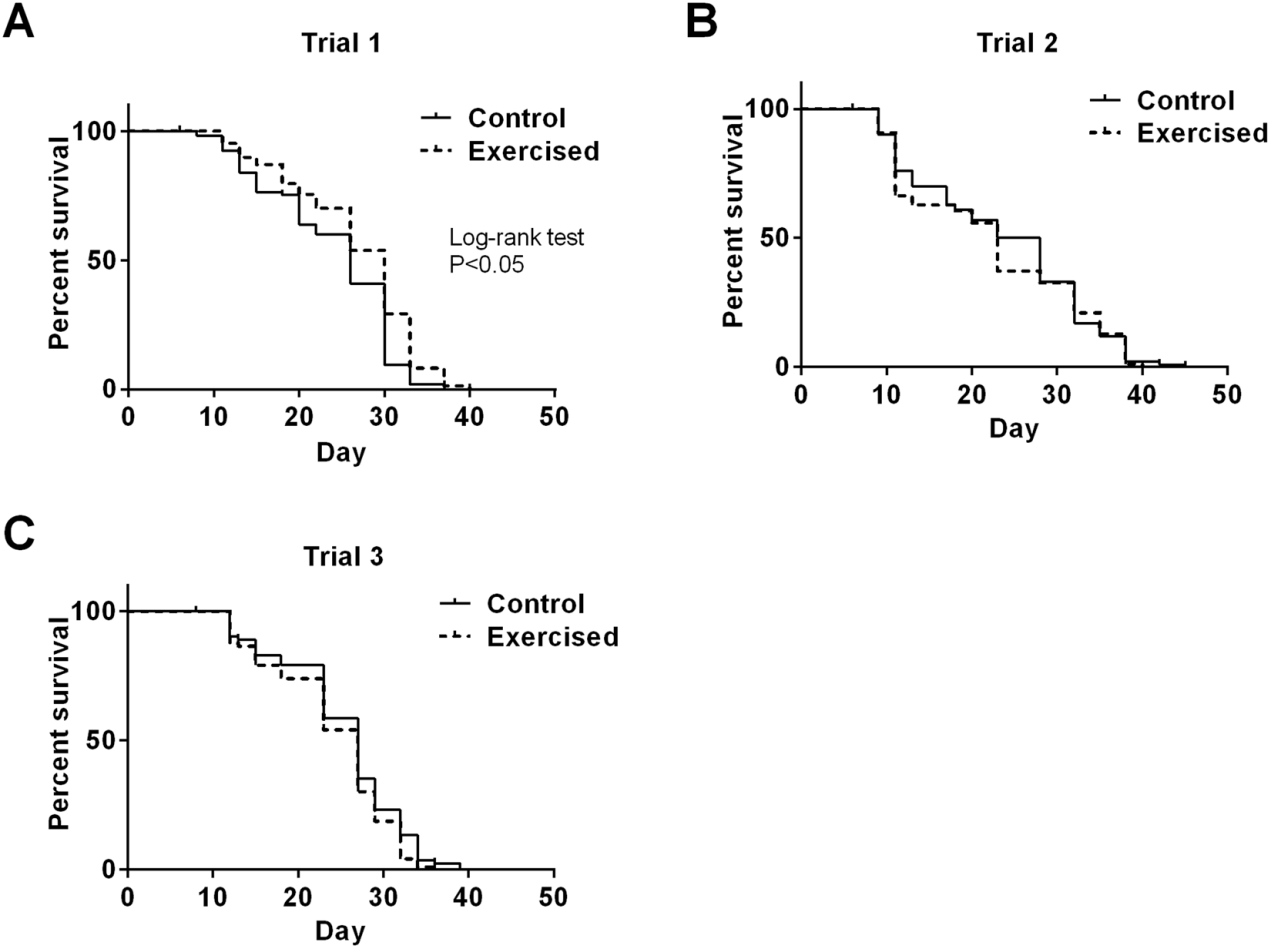
Lifespan curves of three different trials. A significantly greater lifespan extension effect was observed only in the first trial (Log rank test P < 0.05).

Apart from the short-term fitness benefits, we tested whether exercise resulted in any long-term benefits that might be detectable as an extension of lifespan in exercised *C. elegans*. In a total of three independent trials, we only observed a statistically significant lifespan extension effect in one trial. In this one trial, median lifespan of the exercised animals was significantly higher than the controls (Fig. 3 and Table 1), but this effect was not consistent as we did not observe significant lifespan benefits in two subsequent trials. Nonetheless, though the vibration-induced exercise paradigm did not deliver a consistent lifespan enhancement in *C. elegans*, the fitness benefits it conferred suggest that this simple paradigm can stimulate mild exercise benefits in *C. elegans*.

**Table 1:** Survival analysis of the control and exercised animals.

Trial No	N number		Median Lifespan/day		
	Control	Exercised	Control	Exercised	log rank test P value
1	105	147	**26**	**30**	**P < 0.005**
2	100	86	25.5	23	P > 0.05
3	82	96	27	27	P > 0.05

A significant lifespan extension effect was observed only in one of the three-lifespan trials.

### Higher mitochondrial ROS level but lack of mitochondrial adaptations observed in exercised animals

**Figure 4:**
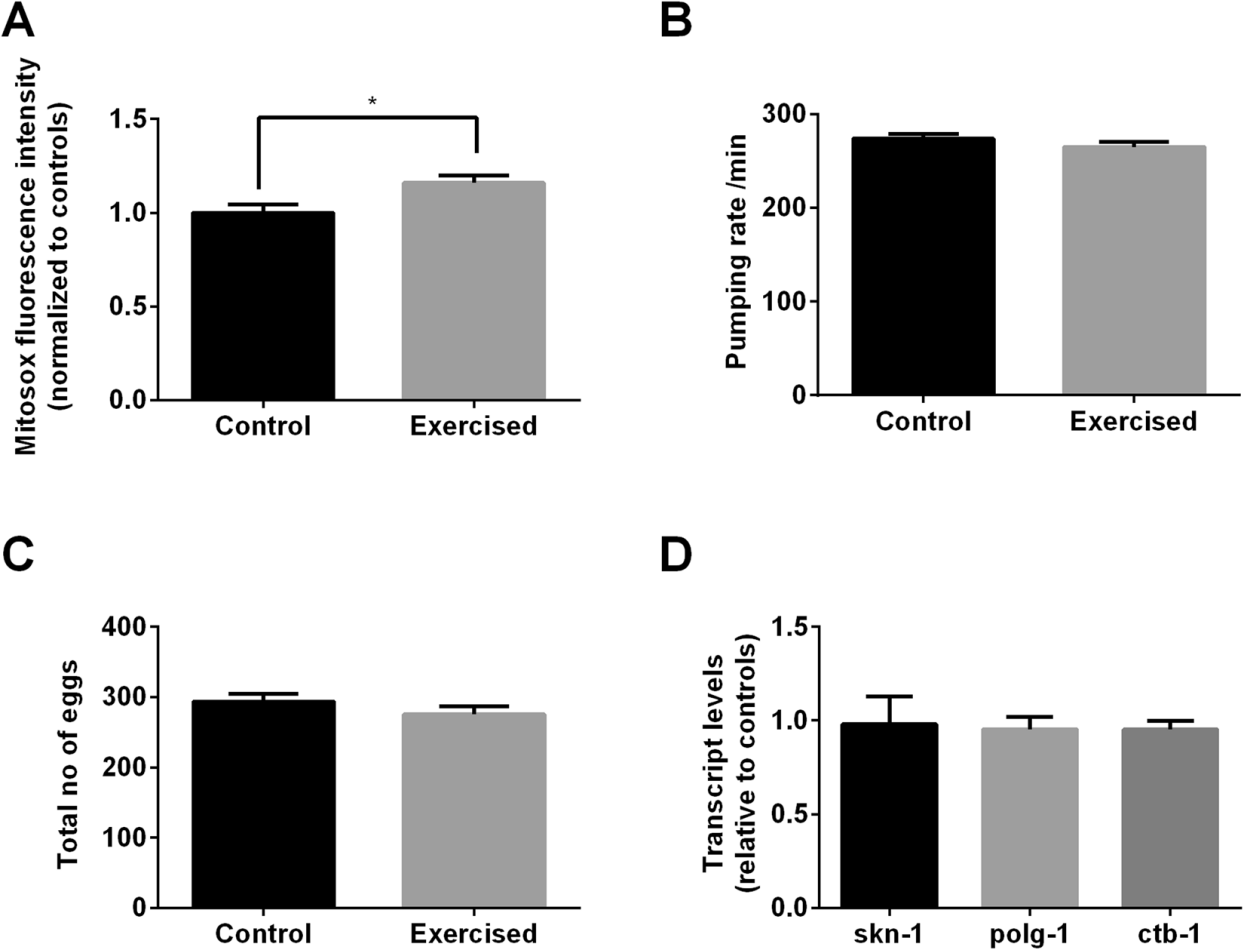
Vibration-induced exercise paradigm increased mitochondrial ROS without inducing toxicity. (**A**) Mitosox fluorescence intensity was significantly higher in the exercised animals compared to controls immediately after the exercise training on day 6 of age (*P < 0.05; n ≥ 25 animals per group). (**B**) Pumping rate, measured immediately after the exercise training on day 6 of age, was not significantly different between the exercised and control animals (n ≥ 15 animals per group). (**C**) Total no of eggs laid was not significantly different between the exercised and control animals (n = 8 animals per group). (**D**) No significant differences were observed for transcript levels of mitohormetic or mitochondrial-related genes *skn-1, polg-1 and ctb-1* between the exercised and control animals one day post exercise (day 7 of age) (n = 3 biological replicates per group; each replicate contains approximately 500 animals collected on different trials). *pmp-3* was used as endogenous control. All experiments described in Fig. 4A–C have been carried out in at least two other trials, with similar trends observed.

According to the mitohormesis theory of exercise, mild levels of mitochondrial stress, induced by reactive oxygen species (ROS) generated during exercise, are required for exercise benefits and this has led to the suggestion that ROS may be key mediators of some exercise benefits^36^. According to the mitohormesis theory, short-term elevation of oxidative stress leads to persistent increases in stress defence and repair and this is the basis of subsequent health benefits^37,38^. To test if exercise at the level resulting from our exercise paradigm indeed resulted in increased ROS production, we measured ROS levels in exercised animals immediately after they underwent the vibration-induced exercise regimen using the MitoSox fluorescence dye. It should be noted that a limitation of this assay is that MitoSox-derived fluorescence is dominated by the pharynx, which may not be representative for the entire animal. Nonetheless, we found that the exercised animals showed significantly higher ROS levels compared to the controls (Fig. 4A). To confirm that this stress level was not harmful to the animals, we measured several parameters of normal physiological functions including egg laying and pharyngeal pumping and found no differences in these behaviours (Fig. 4B and C). These observations suggest that even though there is a significant increase in mitochondrial ROS levels, this increase did not induce overt toxicity to the animals. To further investigate whether the increase in mitochondrial ROS levels triggered hormetic adaptive effects at a transcriptional level, we performed reverse-transcription polymerase chain reaction (RT-PCR) on several ROS and mitohormesis-related genes. We tested for transcriptional changes in *skn-1* (a gene involved in oxidative stress regulation and response*), polg-1* (a gene involved in mtDNA replication) and *ctb-1* (a gene coding for cytochrome b; as a measure of overall mitochondria transcript level). However, we found no differences in expression levels between the exercised and control animals (Fig. 4D), suggesting that the increase in mitochondrial ROS, though not overtly toxic, was not in the hormetic range to trigger gene expression changes sufficiently robust to detect using this method.

**Figure 5:**
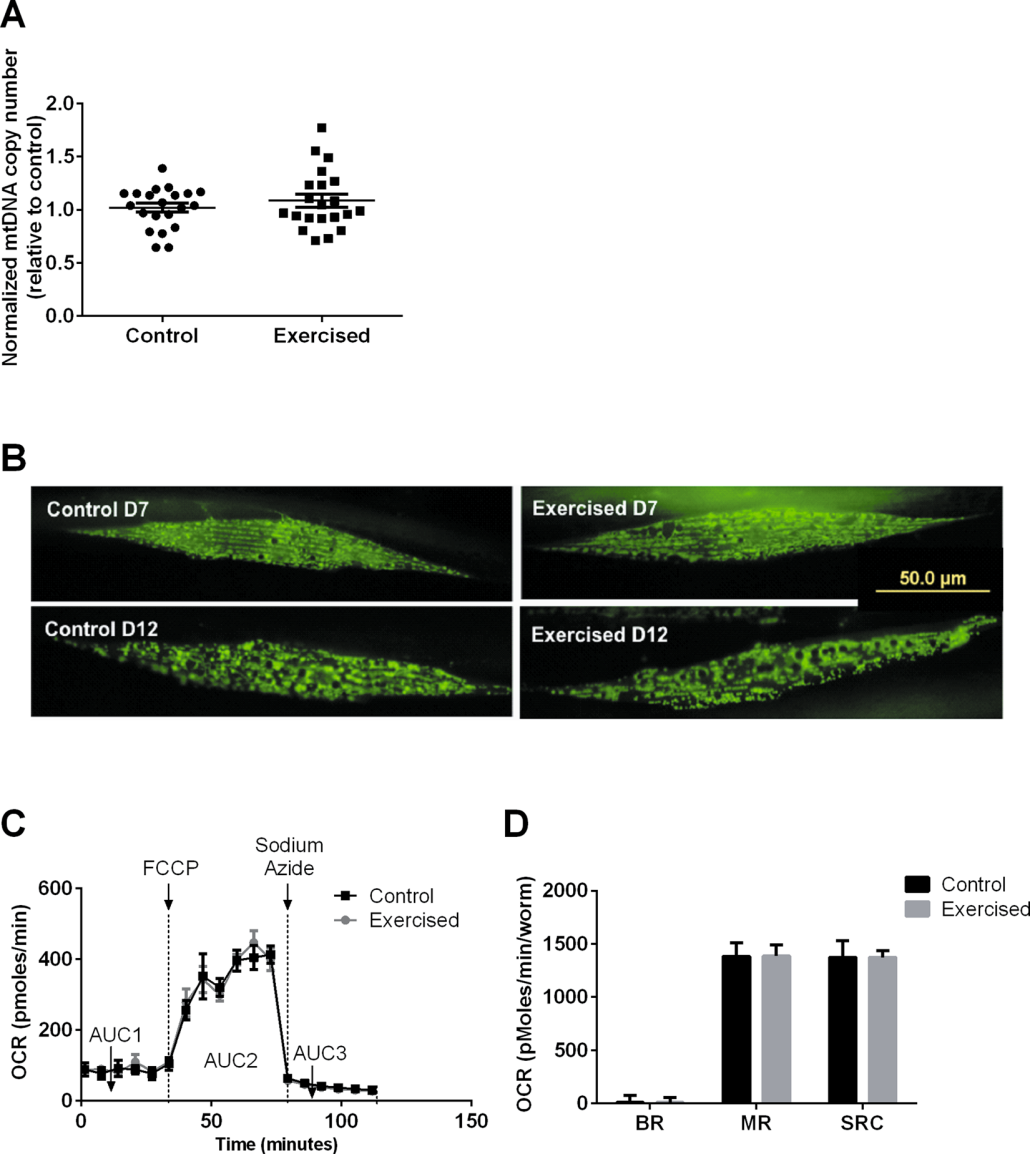
The vibration-induced exercise paradigm did not trigger mitochondrial adaptations in *C. elegans*. (**A**) No significant difference was observed for mitochondrial DNA copy number between the exercised and control animals (n ≥ 20 animals per group). Similar trend was observed in two other trials. (**B**) Representative images of mitochondrial morphology in sarcomeres of young (D7) and old (D12) exercised and control animals, using transgenic strain SJ4103[*myo-3p*::GFP(mit)] (n ≥ 10 animals per group). Similar trend was observed in two other trials. (**C**) Oxygen consumption rate (OCR) profiles of the exercised and control animals measured by Seahorse analyser. AUC1: Area under curve (AUC) of the OCR-time graph of the first six time points; AUC2: AUC of the 7^th^ to 12^th^ time point; AUC3: AUC of the 13^th^ to 18^th^ time point. (**D**) No significant differences were observed for Basal Respiration (BR), Maximal Respiration (MR) or Spare Respiratory Capacity (SRC) between the exercised and control animals (n = 6 repeats per group; each repeat contains 10 animals). BR = AUC3 – AUC1; MR = AUC2 – AUC3; SRC = AUC2 – AUC1. Experiments in this panel were done one day post-training on day 7 of age.

Adaptive exercise benefits in muscle have been reported to involve mitochondrial and metabolic adaptations^39,40^. To test if we could detect exercise benefits at the level of these parameters we measured mitochondrial DNA copy number, observed mitochondrial sarcomere morphology and determined oxygen consumption parameters related to metabolism, as indicators of those adaptations. However, no significant differences in mitochondrial DNA copy number were found between the exercised and control animals (Fig. 5A). We employed a transgenic strain SJ4103[*myo-3p*::GFP(mit)] to visualize mitochondrial sarcomere morphology. This strain expresses GFP in the mitochondria of body wall muscle cells^26^. However, no observable differences in sarcomere mitochondrial morphology were seen between exercise and control groups using this method (Fig. 5B). Metabolic flux was examined using a Seahorse bioanalyzer, using Carbonyl cyanide 4-(trifluoromethoxy) phenylhydrazone (FCCP), a mitochondrial uncoupler, and Sodium Azide, a mitochondrial complex IV inhibitor (see^41,42^ for detailed explanations). Again, no differences in basal respiration (BR), maximal respiration (MR) or spare respiratory capacity (SRC) were observed between the exercised and control animals (Fig. 5C and D). Together these data suggest that despite the training benefits in terms of increased physical performance, adaptations in terms of muscle morphology, mitochondrial number and gross metabolism were absent, or too subtle to be detected using these methods.

### Exercise reduced total Triacylglycerides (TAG) content and its peroxidation index in *C. elegans*

**Figure 6:**
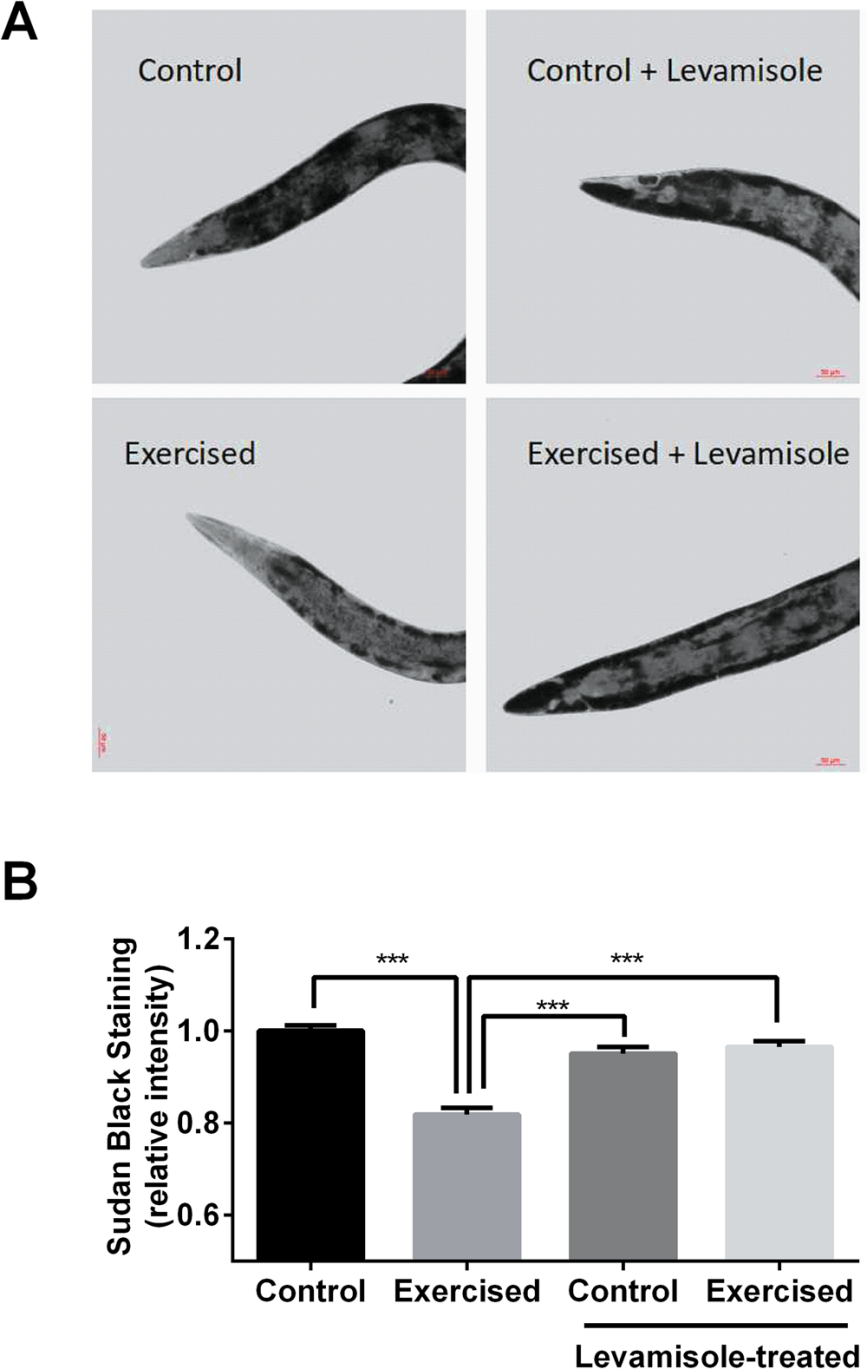
Sudan Black staining of animals. (**A**) Representative images of animals stained with Sudan Black from three independent trials with n ≥ 20 animals per group (scale bar = 50 um). (**B**) Relative Sudan Black staining intensity quantified using ImageJ (One-way ANOVA Sidak’s multiple comparisons post-test analysis ***P < 0.001; n ≥ 20 animals per group). Similar trend was observed in two other trials.

Exercise has been known to induce fatty acid oxidation resulting in weight loss and changes in lipid profiles in humans^43,44^. To study the effect of exercise on *C. elegans’* lipid storage, we first measured total lipid content using Sudan Black, a lipophilic dye that stains neutral Triacylglycerides (TAG), a major class of storage lipids^34^. Using this approach, we found that the exercised animals had a lower TAG content than the controls (Fig. 6A and B). Animals paralyzed by levamisole treatment did not show any reduction in TAG content whether they were subjected to vibration or not, hence suggesting that the lipid reduction benefit was mediated by exercise (enhanced activity) and not due simply to mechanical stimulation or starvation (Fig. 6A and B).

**Figure 7:**
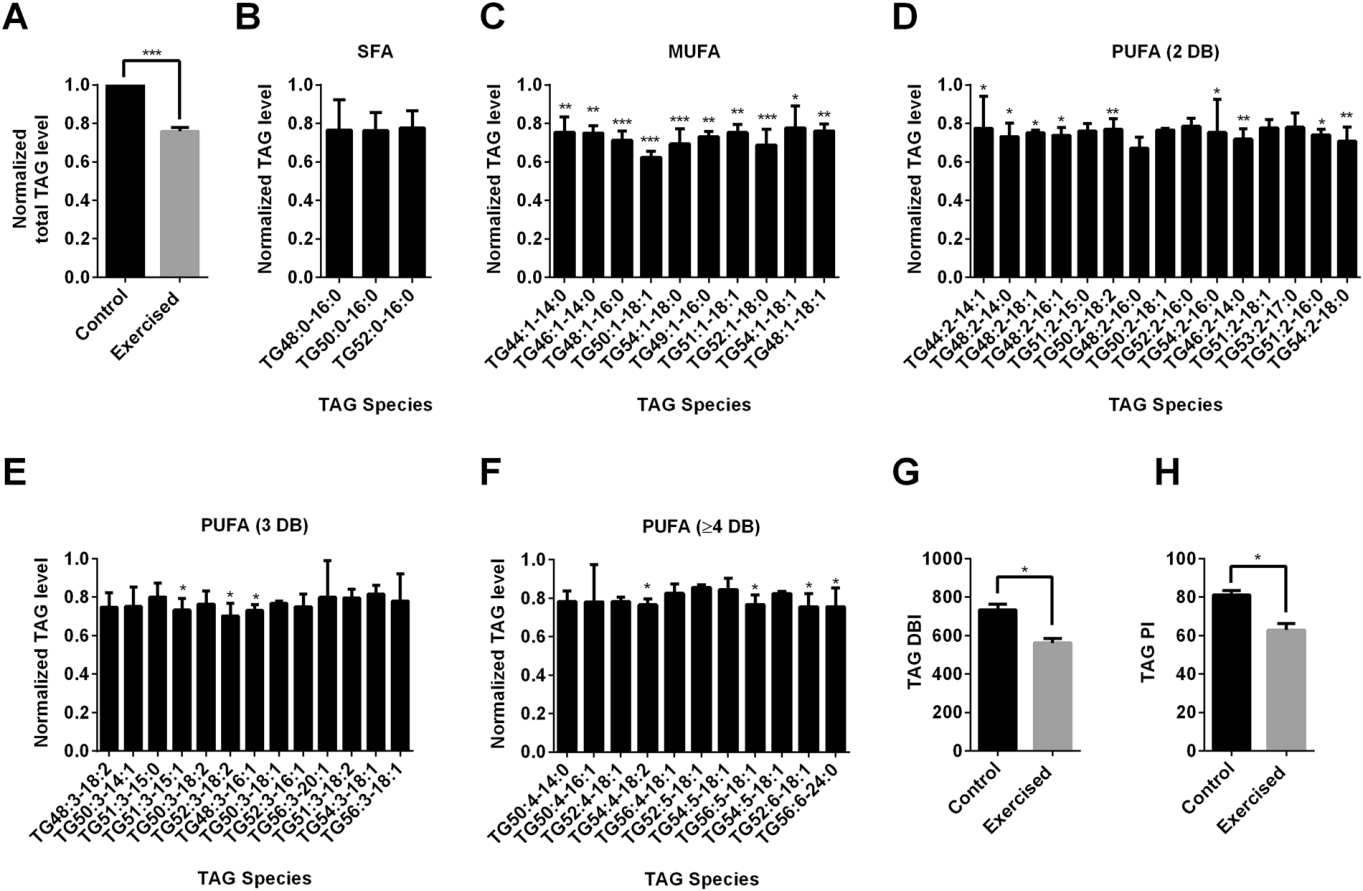
Exercise modulated lipid metabolism in *C. elegans*. (**A**) Representative images of control and exercised animals stained with Sudan Black (n ≥ 25 animals per group; scale bar = 200 um). Similar trend was observed in two other trials. (**B**) Total TAG level was normalized to controls and was significantly lower in the exercised animals than the controls (***P < 0.001; n = 3 repeats per group; each repeat contains approximately 2000 animals collected from different trials). (**C**–**G**) TAG species profile of the exercised animals normalized to controls (Two-way ANOVA for strain P < 0.001; post-test Sidak’s multiple comparisons analysis *P < 0.05, **P < 0.005, ***P < 0.001; n = 3 repeats per group; each repeat contains approximately 2000 animals collected from different trials). (**G**) TAG Double Bond Index (DBI) was significantly lower in the exercised animals than the controls (*P < 0.05; n = 3 repeats per group; each repeat contains approximately 2000 animals collected from different trials). (**H**) TAG Peroxidation Index (PI) was significantly lower in the exercised animals than the controls (*P < 0.05; n = 3 repeats per group; each repeat contains approximately 2000 animals collected from different trials). SFA: Saturated Fatty Acids; MUFA: Mono-unsaturated Fatty Acids, PUFA: Poly-unsaturated Fatty Acids; DB: Double Bond. Experiments in this panel were done immediately after the exercise paradigm on day 6 of age.

To further confirm the differences in TAG levels, we performed liquid chromatography-mass spectroscopy (LCMS) analysis and found a ~25% reduction in the total TAG levels in the exercised animals compared to the controls (Fig. 7A). We then carried out a full lipidomics analysis to determine the identity of lipid species most affected. Amongst the individual TAG species, no significant differences were observed within the saturated fatty acid (SFA) residues, while significant reductions in various mono-unsaturated fatty acid (MUFA) and poly-unsaturated fatty acid residues (PUFA) species were seen in the exercised animals compared to the controls (Fig. 7B–F). This trend suggests that exercise alters the degree of lipid unsaturation of TAG.

The degree of lipid unsaturation is related to its vulnerability to lipid peroxidation, as the presence of double bonds in MUFA and PUFA make them more vulnerable to peroxidation than SFA^45^. Double Bond Index (DBI) and Peroxidation Index (PI) of lipids have been suggested as indicators of lipid peroxidation susceptibility. DBI refers to a simple weighted average of the number of double bonds per fatty acid molecule while PI reflects the susceptibility of a class of lipid to peroxidation by considering the weighted proportion of MUFA and PUFA^46^. Higher DBI or PI indicates greater vulnerability to lipid peroxidation, and consequently is related to oxidative damages that are detrimental to health- and lifespan^47^.

To determine whether the lipid changes observed in the exercised animals significantly affected these indicators of susceptibility to lipid peroxidation, we calculated the DBI and PI of TAGs for exercised and control animals. We found that both DBI and PI were significantly lower in the exercised animals than the controls (Fig. 7G and H). These lower indices suggest a protective lipid remodelling effect induced by exercise that resulted in a lower lipid susceptibility to peroxidation.

## Discussion

We sought to determine whether *C. elegans* can be made to exercise on solid agar plate, using a simple vibration-induced paradigm. After a series of careful optimization experiments, we successfully developed an interval-training regimen using the vibration paradigm to stimulate exercise in *C. elegans*. This novel exercise paradigm improved physical and neuronal fitness, and modulated lipid storage and storage lipid composition in *C. elegans*. Despite a significant increase in mitochondrial ROS production, no evidence for damage or mitohormetic adaptations, at least in gene expression, were observed in the exercised animals, and no consistent lifespan benefit was found in the exercised animals. The exercise paradigm was performed using JK1107 animals which do not produce a germline. This sterile mutant was chosen to avoid confounding effects of oocytes during mitochondrial assays and lipid analysis. Given that germline exerts physiological responses that can impact aging quality^48^, further studies should investigate the role of germline signalling in exercise benefits during ageing.

Our finding that a simple exercise paradigm in *C. elegans* can deliver similar benefits observed in humans is in agreement with recent advances in the emerging *C. elegans* exercise literature. Using an electrotactic flow chamber to exercise *C. elegans* through swimming, Chuang *et al*. showed that this form of exercise similarly reduced lipid content and enhanced antioxidant defence system of nematodes, and prevented sarcomere and mitochondria degeneration in aged nematodes^26^. Laranjeiro *et al*. further showed that a single 90 min swim session in M9 buffer was able to trigger immediate gene expression changes (within 2 h of swim exercise) that result in strengthening of oxidative stress defence system (upregulation of superoxide dismutase *sod-4* and *sod-5*)^27^. Unlike these papers, however, we did not find a protective mitochondrial nor mitohormetic effect with a single 6.5 h exercise programme. We looked at gene expression changes one day post exercise while Laranjeiro *et al*. examined the transcriptomic changes immediately after the swim training. Given the short lifespan of *C. ele*gans (~20 days) this may be too long an interval after training to observe transcriptional responses. The difference in time point may explain the lack of protective effect observed in our study, as Laranjeiro *et al*. reported that the protective effect of exercise against juglone stress was no longer observed 24 h post swim training. Moreover, in contrast to the other two forced swim paradigms in *C. elegans* which involve greater energy expenditure than crawling activity^27^, the intensity of our exercise paradigm is milder. This difference in exercise intensity likely explains the lack of mitochondrial adaptation observed in our exercised animals.

Nonetheless, in agreement with the other two exercise paradigms, we have shown that, similar to swimming exercise, vibration-induced exercise reduces fat storage and significantly alters lipid composition of *C. elegans*. Our finding of a reduction in TAG levels post-exercise is also consistent with Laranjeiro *et al.’s* findings that swim exercise upregulates genes involved in lipolysis, fatty acid activation and beta-oxidation in *C. elegans*. These findings thus suggest a conserved mechanism underlying exercise benefits in *C. elegans* regardless of the paradigm used.

To the best of our knowledge, we are the first group to have extensively characterized the lipid changes mediated by exercise in *C. elegans*. Our observation that the individual TAG species that are significantly reduced in the exercised animals contain MUFA and PUFA but not SFA is in agreement with results from the exercise study carried out by May and colleagues in mice^44^. Importantly, the lower vulnerability to lipid peroxidation of TAG in the exercised animals, as indicated by their lower DBI and PI compared to the controls, suggests that exercise confers protection against harmful radicals and lipid peroxidation, which is one of the main detriments of oxidative stress^47^.

Despite the short-term exercise benefits and changes in lipid composition, we did not observe a consistent lifespan benefit in the exercised animals. However, it should be noted that we did not optimize the exercise regime to maximize lifespan effects and only investigated potential benefits following a single day of training on day six of life. Lifespan is a rather crude measure of health, and whether or not exercise causes lifespan extension is unclear even in humans^49^. While several studies have shown that moderate exercise is associated with lower mortality in humans, mixed findings were reported regarding the lifespan benefits of endurance sports in elite athletes (reviewed in^50^). Similarly, in mice, exercise was found to improve healthspan but not necessarily lifespan^51,52^. Of the other two exercise studies in *C. elegans*, one resulted in extension of median lifespan of exercised N2 (~10% extension) and transgenic CL2120 animals with amyloid-beta expression (~20% extension)^26^ while the other did not report any lifespan study^27^. It therefore appears that exercise may not necessarily lead to consistent lifespan enhancement. Further studies should evaluate the benefit of these exercise paradigms on more subtle markers of healthspan and in *C. elegans* models for metabolic or age-related disease.

In comparison to human exercise, the vibration-induced exercise paradigm resembles whole body vibration (WBV) training in humans. WBV is a passive form of exercise where mechanical stimulation of skeleton is achieved by having a person standing on a vibration plate^53^. WBV training has been shown to improve physical fitness, bone mass, muscle strength and mobility in elderly via neuromuscular adaptations^54–59^. Even though the vibration-induced exercise paradigm reported in our paper is not exactly a passive exercise as it stimulates active movement of the animals, we found similar benefits of improved fitness and mobility in the exercised nematodes. Future work using our vibration-induced exercise paradigm on aged nematodes may provide insights on the neuromuscular adaptations underlying WBV benefits in elderly.

In summary, the creation of an exercise paradigm in *C. elegans* provides new tools and opportunities for researchers to expand our knowledge on exercise. The emergence of different exercise paradigms in *C. elegans* as well as other small invertebrate model organisms allows us to understand the molecular mechanisms underlying exercise benefits, which may in turn aid and accelerate the discovery of exercise mimetics for healthspan interventions.

## Materials and Methods

### Nematode strains and maintenance

The temperature-sensitive sterile mutant JK1107[*glp-1*] was used in all experiments, except in the egg-laying experiment which used the Bristol N2 strain, and the mitochondrial morphology visualization which used the SJ4103[*myo-3p*::GFP(mit)] strain. JK1107 animals were raised at the permissive temperature of 16 °C and cultivated at the restrictive (sterility inducing) temperature of 25 °C post-bleaching to prevent progeny production. All other strains were raised and cultivated at the standard temperature of 20 °C. Animals were grown on nematode growth medium (NGM) agar plates prepared as described in^60^. All experiments used age-synchronized animals obtained *via* hypochlorite bleaching using standard protocol^61^. All strains were obtained from the *Caenorhabditis* Genetics Centre (CGC).

### Exercise paradigm

Unless otherwise stated, the animals underwent the exercise training on day 6 of age (post-bleaching) in an incubator maintained at 25 °C. Day 6 was chosen as exercise training at this age produced the greatest training effect in terms of the relative increase in travel speed during the vibration protocol (Fig. S2). The exercise paradigm also resulted in benefits later in life (day 8), but both speed of movement prior to training and training benefits were smaller (Fig. S2).

Mechanical stimuli, in the form of vibration, were transduced to the animals on NGM agar plates using a system of 4 audio speakers. Each agar plate was placed on top of a separate speaker (FR 10 HM - 8Ω; Visaton, Germany). The speakers were connected to a function generator (TG550; Aim-TTi, UK), audio amplifier (AI-2210; Sherwood, USA) and time sequencer (TSC001; Thorlabs, USA) as shown in Fig. 1A. The peak-to-peak voltage and current across each speaker were 5.6 V and 800 mA, respectively. The time sequence of stimulation is shown in Fig. 1E.

For levamisole treatment, JK1107 animals raised on 25 °C were transferred to NGM plate containing 1 mM levamisole hydrochloride (Sigma-Aldrich, St. Louis, United States) 30 min before the exercise paradigm. Immediately after the exercise paradigm, the levamisole-treated animals were transferred to a fresh NGM plate for recovery.

### Speed calculation

To calculate speed of movement immediately after exercise 30 s videos of animals moving freely on NGM plates were captured using a 16 MegaPixel digital camera (DV150F; Samsung, South Korea). Trajectories were analysed using a Matlab programme (written in-house) that allows their speed to be calculated based on waypoints along the trajectory.

### Fitness assays

Both spontaneous distance travel and swimming abilities were measured one day after the end of exercise training. For the spontaneous distance travel assay, animals were transferred to a fresh NGM plate without any food. Five minutes after the transfer, one-minute videos of animals moving freely on the NGM plate were captured using a 16 MegaPixel camera (DV150F; Samsung, South Korea). Trajectories were analysed using our Matlab programme that allows their total distance travelled, peak speed, time spent actively foraging and percentage non-exploring nematodes to be calculated. Actively foraging behaviour was defined as moving more than 0.4 mm in a five-second interval while percentage non-exploring animals was defined as those that stayed around the origin and moved less than 5 mm total distance during the one-minute interval. For the swimming assay, animals were transferred to M9 buffer in a 96-well plate at a density of 10 animals per well. Two hours after the transfer, the percentage of actively thrashing nematodes was calculated. Both assays were conducted at room temperature (20 °C).

### Lifespan assay

Animals were transferred to fresh NGM plate at a density of 50 animals/plate one day post-exercise with 200ul bacteria/plate. No further transfer was done in subsequent days as this amount of bacteria was sufficient to maintain the animals for the entire lifespan assay. The number of dead animals (those that failed to respond to mechanical touch) was established every 1 to 3 days, depending on prevalent mortality. All lifespan trials were scored under observer-blinded condition to minimize observer bias^62^. Lifespan assay was conducted at 25 °C.

### Pumping assay

Pumping assays were conducted immediately after the exercise training to show that the vibration paradigm did not cause the animals to stop pumping. One-minute videos of animals were recorded at 80X magnification using a dissecting microscope (MZ16; Leica, Singapore). The number of pharyngeal pumps performed per minute was quantified by viewing the videos in slow motion, at room temperature (20 °C).

### Egg-laying assay

For egg-laying assay, N2 animals were used and underwent the exercise training on day 3 of age post-bleaching. N2 animals were exercised on day 3 instead of day 6 as egg-laying activity peaks on day 3. Animals were then placed singly on a 3 cm NGM plate seeded with bacteria during the exercise regimen, and transferred to a fresh 3 cm seeded NGM plate daily until day 7 post-bleaching. The number of progenies on each plate was counted 2 days after egg-laying and summed across the days to obtain the total number of eggs per animal. This assay was conducted at room temperature (20 °C).

### ROS assay

Day 5 animals (post-bleaching) were grown on NGM plates supplemented with 10 uM MitoSOX (Molecular Probes, Singapore). Following 36 h incubation at 25 °C, animals were transferred to a fresh NGM plate and subjected to the exercise regimen or left to roam freely (control). Immediately after exercise, animals were transferred to a microscope slide containing 18 ul of M9 buffer. Images of the pharynx were acquired using Olympus IX81 microscope (Olympus, Tokyo, Japan), with WG filter cube (Excitation Filter BP510-550). Fluorescent images were captured under 20X magnification, using a Xenon arc lamp. Bright field and fluorescent images were superimposed at a 30:70 ratio, and the relative fluorescence intensity was measured using ImageJ (NIH, Bethesda, USA).

### Mitochondrial assays

Mitochondrial DNA copy number of animals was quantified as described in^60^ using a real-time PCR method. Mitochondrial morphology was visualized under the Olympus IX81 microscope (Olympus, Tokyo, Japan), using SJ4103[*myo-3p*::GFP(mit)], a transgenic strain expressing GFP in the mitochondria of body wall muscle cells. Oxygen consumption profile related to metabolic parameters was measured using XF96 Extracellular Flux Analyzer (Seahorse Bioscience, USA), as described in^42^.

### RNA extraction and RT-PCR

Total RNA samples of animals were collected from approximately 500 animals per repeat for RT-PCR and RNAseq. Total RNA was extracted using the RNeasy Micro Kit (Qiagen, Netherlands) while reverse-transcription was performed using GoScript Reverse Transcription System (Promega, USA), as per manufacturer’s instructions. PCR was performed using SyBr system as described in^41^. Primer sequences used were shown in Table 2.

**Table 2:** Primer sequences used for RT-PCR.

Transcript name	Primer sequences
*skn-1*	FP: GTTCCCAACATCCAACTACG RP: TGGAGTCTGACCAGTGGATT
*polg-1*	FP: CTGCCTAATACCGTTGCCTTCTT RP: TTGGAGCCGTCCGGATT
*ctb-1*	FP: TTCCAATTTGAGGGCCAACT RP: AACTAGAATAGCTCACGGCAATAAAAA
*pmp-3*	FP: TGGCCGGATGATGGTGTCGC RP: ACGAACAATGCCAAAGGCCAGC

FP: Forward Primer; RP: Reverse Primer.

### Sudan Black staining

Sudan black staining was performed on animals one day after the exercise regimen, as described in^34^. Relative staining intensity was quantified using ImageJ.

### Lipid extraction

Approximately 2000 nematodes per sample were collected, washed with M9 buffer and transferred to 2-mL polypropylene tubes containing 250 µl lysis buffer (20 mM Tris-HCl pH 7.4, 100 mM NaCl, 0.5 mM EDTA, 5% glycerol) and left on ice for 15 minutes followed by homogenization using a Bead beater (Omni International, USA) maintained at 4 °C. Lipids extraction from the lysed samples was carried out by Folch’s extraction^63^. In order to minimize oxidation during and after extraction, 0.5% butylated hydroxytoluene (BHT) was added to the organic solvents. For method validation, samples were spiked with known amounts of internal standards (purchased from Avanti polar lipids, Alabaster, AL, USA) corresponding to each lipid class during the single-phase extraction to control lipid-class dependent differences in extraction and ionization^64^. Standards used were PC-34:0, PE-28:0, SM-30:1, Cer-35:1, DAG-24:0 and TAG-48:0. To achieve separation into aqueous and organic phases, the samples were vortexed and centrifuged at 3000 rpm for 5 minutes. The lower phase was then transferred to a fresh centrifuge tube and centrifuged in a vacuum concentrator (SpeedVac, Thermo Savant, Milford, USA)) until they were dry. The dried lipid extract was reconstituted in 50 µl methanol and left at −80 °C until the mass spectrometry analysis was performed.

### Mass-spectrometric analysis and data processing

A 6490 triple Quad Mass spectrometer (QqQ; Agilent, USA) coupled to a 1260-Ultra Performance Liquid chromatography (UPLC) was used for lipid quantification. ESI was used to ionize lipids. Each lipid molecular species was analysed using a targeted dynamic multiple reaction monitoring (dMRM) approach containing transitions for known precursor/product mass-to-charge ratio (m1/m3) and retention times. The UPLC system was equipped with a Waters ACQUITY BEH C18column (1.0 × 100 mm) to separate the molecular species using gradient elution. Solvent A was acetonitrile/H2O (60:40) with 10 mM ammonium formate and 1% NH_4_OH, while solvent B was isopropanol/acetonitrile (90:10) containing 10 mM ammonium formate and 1% NH_4_OH. The flow rate was 0.13 mL/min and the column temperature 60 °C. Solvent B was set at 40% at injection and increased linearly to 100% in 14 minutes, retained at this value for 3 minutes, decreased back to 40% in one minute and then retained there until the end of the gradient by 20 minutes. The eluent was directed to the ESI source of the mass spectrometer operated in the positive ion mode. The MS conditions were as follows. For ESI: gas temperature, 300 °C; gas flow, 10 l/minutes; sheath gas temperature, 350 °C; sheath gas flow, 8 l/minutes; and capillary voltage, 3,500 V. For APCI: gas temperature, 300 °C; vaporizer, 450 °C; gas flow, 5 l/minutes; capillary voltage, 4,000 V; and corona current, 4 μA.

High-resolution LC-MS data obtained on the 6490 QqQ Mass spectrometer were subjected to data processing using MassHunter software (Agilent). The identification of a particular species was based on accurate mass and/or retention times (RT). Signal intensities of the targeted species were compared with the intensities from the spiked internal standards and the retention times for the various classes were matched. Data processing, included peak smoothing and integration of areas under the curves for each ion measured. The processed data were exported to excel and normalized to protein content as well as the internal standard. Fold changes were measured by comparison of the different conditions to the control sample and finally the Student’s t test was performed to determine whether differences between the samples were statistically significant. (p < 0.05 was considered statistically significant).

### Statistical analysis

All statistical analyses were conducted in GraphPad Prism version 5.0 (San Diego, USA). Unless otherwise stated, data were analyzed using unpaired t-tests. All data were reported and shown as mean $\pm$ standard error of the mean (SEM).

### Data availability statement

All data generated or analysed during this study are included in this published article (and its Supplementary Information files).

## Electronic supplementary material

Supplementary information
