# Supplementary material for: A novel vibration-induced exercise paradigm improves fitness and lipid metabolism of Caenorhabditis elegans
Source: Sci Rep. 2018 Jun 20;8:9420. doi: 10.1038/s41598-018-27330-3 (PMC6010440; doi:10.1038/s41598-018-27330-3)
Supplement: Supplementary file 1 — Supplementary information [file 41598_2018_27330_MOESM1_ESM.pdf]

Title: A novel vibration-induced exercise paradigm improves fitness and lipid metabolism of *Caenorhabditis elegans*

Author list: Emelyne Teo[1], Krishna Chaitanya[2,3], Diogo Barardo[3,4], Linfan Xiao[4], Amaury Cazenave-Gassiot[2,3], Nicholas Tolwinski[4], Markus Wenk[2,3], Barry Halliwell[3], Jan Gruber[4,3*]

[1]NUS Graduate School for Integrative Sciences and Engineering, National University of Singapore, Singapore [2]Singapore Lipidomics Incubator, National University of Singapore, Singapore [3]Department of Biochemistry, National University of Singapore, Singapore [4]Science Division, Yale-NUS College, Singapore

***Corresponding author; contact email:** jangruber467@gmail.com

Supplementary figures

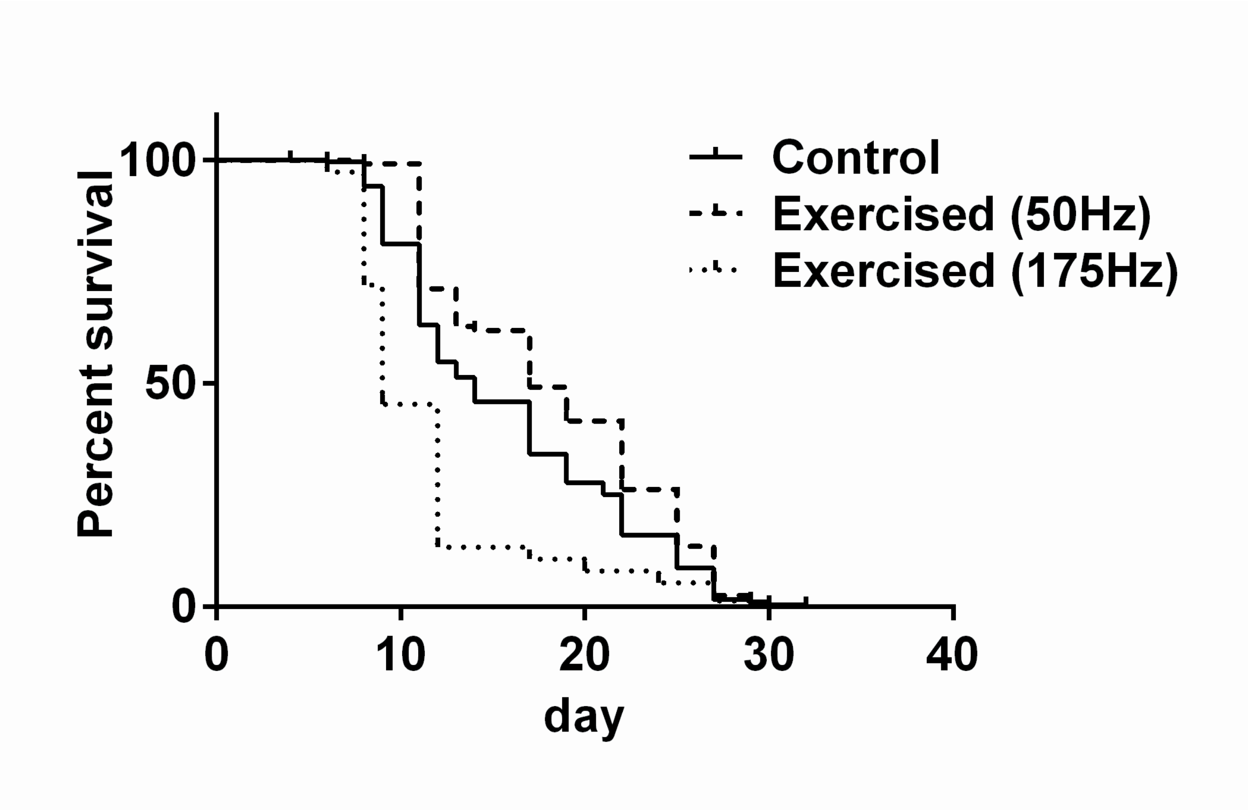

Fig S1. Lifespan curves of animals exercised at different frequency. Lifespan of animals exercised at 175Hz was significantly shorter than the controls (log-rank test P<0.001).

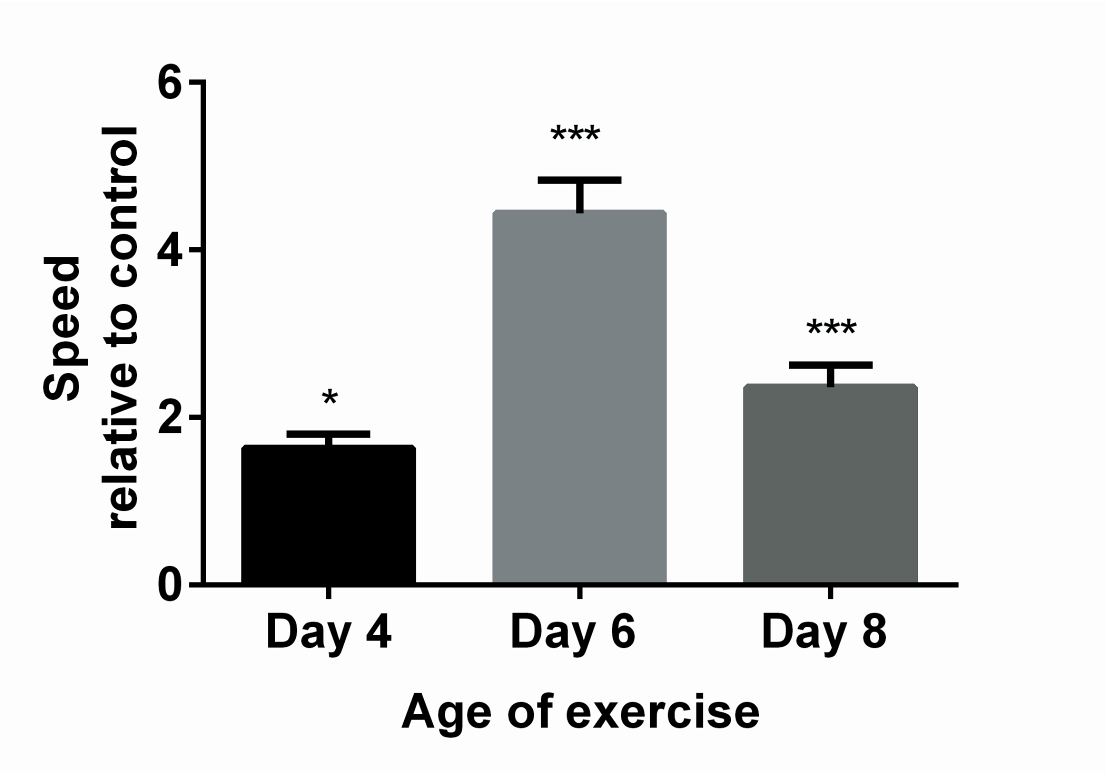

Fig S2. Travel speed of exercised animal during vibration relative to controls. Greatest increase in travel speed during vibration was observed when animals were exercised on day 6 (*P<0.05, ***P<0.001; n=8 animals per group).

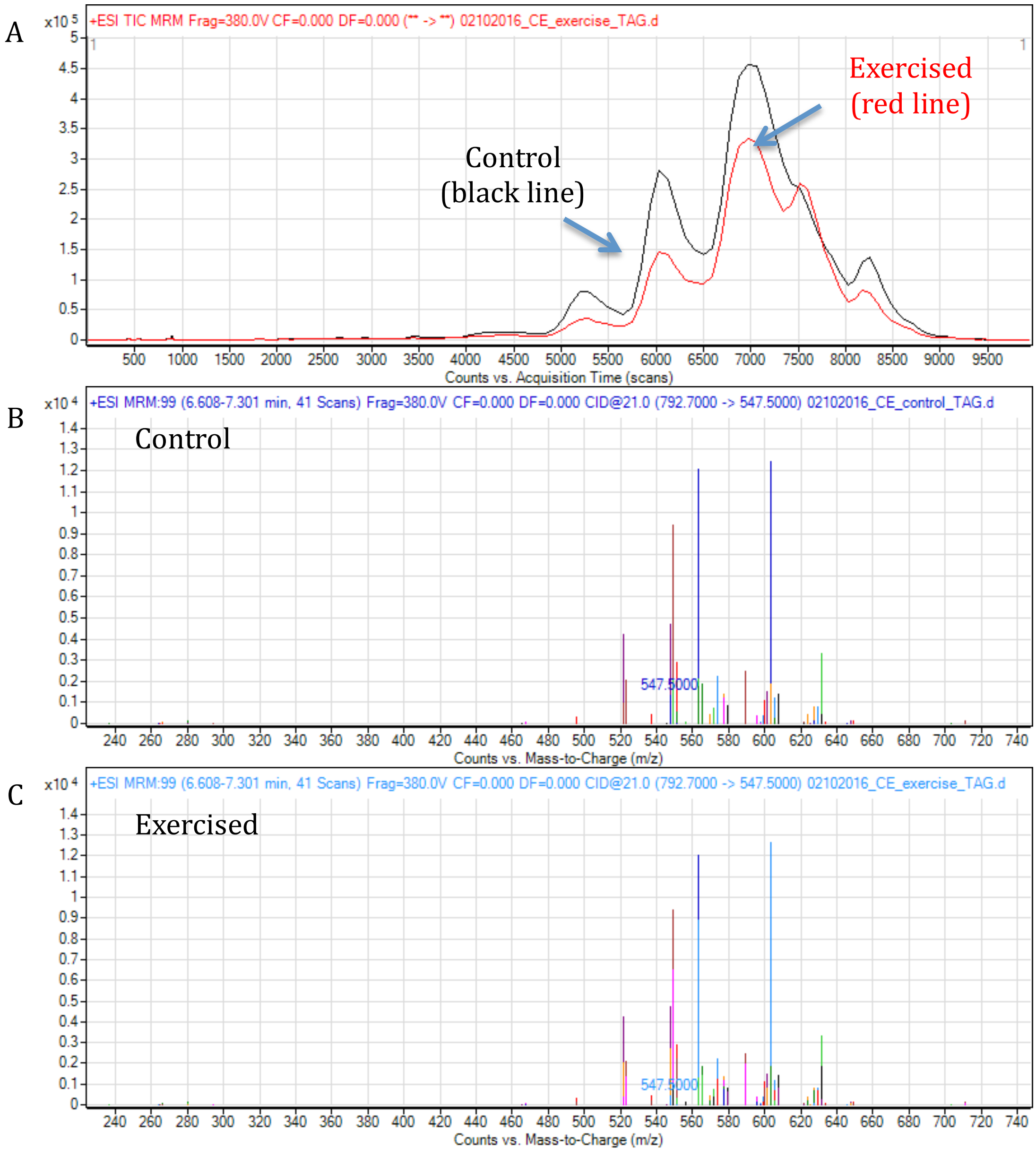

Fig S3. LC-MS data. A) Total Ion Chromatograms (TIC) of Triacylglycerols (TAGs) detected by employing dynamic multiple reaction monitoring (dMRM) methods. The representative overlapping chromatograms showing LC separation of TAGs from *C. elegans* control and exercised lipid extracts (black trace line vs. red trace line). A significant decrease in the TAG levels can be noticed. Here the x-axis is the total acquisition time (0 min to 11 min) that shows the retention time of molecular species monitored for while the y-axis represents the total ion count. B) MS spectra extracted from Total Ion Chromatograms (TIC) of TAGs are shown here. The top panel represents the MS spectrum extracted from the control *C.elegans*lipid extracts while the bottom panel is from the lipid extracts from the exercised animals. The MS spectra represents TAG species counts between 450 m/z and 950 m/z.
